# Imperatorin's Effect on Myocardial Infarction Based on Network Pharmacology and Molecular Docking

**DOI:** 10.1155/cdr/7551459

**Published:** 2025-01-13

**Authors:** Ruizhe Zhang, Peng Wang, Yao Jin, Qingya Xie, Pingxi Xiao

**Affiliations:** ^1^Department of Cardiology, Sir Run Run Hospital, Nanjing Medical University, Nanjing, China; ^2^Key Laboratory of Cardiovascular and Cerebrovascular Medicine, School of Pharmacy, Nanjing Medical University, Nanjing, China; ^3^Department of Cardiology, The Fourth Affiliated Hospital, Nanjing Medical University, Nanjing, China

**Keywords:** ACE–Ang II–AT1R axis, imperatorin, molecular docking, myocardial infarction, network pharmacology

## Abstract

**Purpose:** Myocardial infarction (MI), a severe cardiovascular disease, is the result of insufficient blood supply to the myocardium. Despite the improvements of conventional therapies, new approaches are needed to improve the outcome post-MI. Imperatorin is a natural compound with multiple pharmacological properties and potential cardioprotective effects. Therefore, this work investigated imperatorin's therapeutic effects and its mechanism of action in an MI mouse model.

**Methods**: Network pharmacology, molecular docking, and experimental validation were performed for exploring the pharmacokinetic properties, therapeutic effects, and molecular targets of imperatorin in MI. Permanent ligation of the left anterior descending artery was performed in male C57BL/6 mice to induce MI before treatment with imperatorin once per day for 1 week. Echocardiography, heart histology, RNA sequencing, and quantitative reverse transcriptase polymerase chain reaction (qRT-PCR) as well as western blotting were carried out for evaluating cardiac function and structure, as well as gene and protein expression.

**Results**: Imperatorin significantly improved cardiac function, preserved cardiac structure, attenuated cardiac remodeling and fibrosis, and reduced cardiomyocyte apoptosis in MI mice. Eight differentially expressed genes overlapping with key target genes were found, two upregulated and six downregulated. A key target in signaling pathways associated with imperatorin effect in MI was angiotensin-converting enzyme (ACE). Imperatorin inhibited ACE–angiotensin II (Ang II)–angiotensin II Type 1 receptor (AT1R) axis in MI mice.

**Conclusion**: Imperatorin attenuated MI by inhibiting the ACE–Ang II–AT1R axis. Thus, imperatorin might be considered a potential therapeutic agent to cure MI.

## 1. Introduction

Myocardial infarction (MI), a severe cardiovascular disease, occurs due to coronary artery obstruction which leads to an insufficient blood supply to the myocardium [1]. Globally, MI remains a leading cause of mortality, significantly impacting an affected individual's disease prognosis and quality of life [2]. Cardiovascular diseases are responsible for approximately 17.9 million deaths each year, accounting for 31% of all global deaths, with a significant proportion of these fatalities due to MI [3]. Moreover, data from the Global Burden of Disease Study indicate an increasing trend in MI prevalence, particularly in low- and middle-income countries, underscoring the urgent need for effective therapeutic strategies [4].

The management of MI has significantly evolved over the years by performing timely reperfusion therapies, including thrombolytic therapy and percutaneous coronary intervention, all reducing myocardial damage and improving patient outcomes [5]. Furthermore, pharmacological interventions, namely, angiotensin-converting enzyme (ACE) inhibitors, beta-blockers, and antiplatelet agents, are effective in secondary prevention and mitigation of adverse cardiac events post-MI [6]. Despite the improvements of conventional therapies, new and complementary approaches are needed to alleviate myocardial damage and improve outcomes post-MI, which are the most relevant fields of research. Natural products possess diverse pharmacological effects due to the presence of multiple bioactive compounds, along with their common interaction with several biological targets, holding broad prospects as new drugs [7].

Imperatorin is a natural furanocoumarin compound derived from various plant sources, such as *Foeniculum vulgare* Mill, *Rhodiola bupleuroides*, *Angelica sinensis*, *Notopterygium incisum*, and *Angelica dahurica*. It gained attention due to its versatile reported pharmacological characteristics, including anti-inflammatory, antioxidant, antitumor, and cardioprotective effects [8, 9]. Recent studies demonstrated that imperatorin acts as a calcium antagonist, reducing blood pressure and inhibiting myocardial hypertrophy in spontaneously hypertensive rats [10, 11]. In patients with renal injury associated with renovascular hypertension, imperatorin exerts both antioxidant and antihypertensive effects [12]. Beyond these studies, current studies are focused on the antitumor mechanisms of imperatorin; yet, the potential effects of imperatorin on MI and associated molecular mechanism are poorly understood.

**Figure 1:**
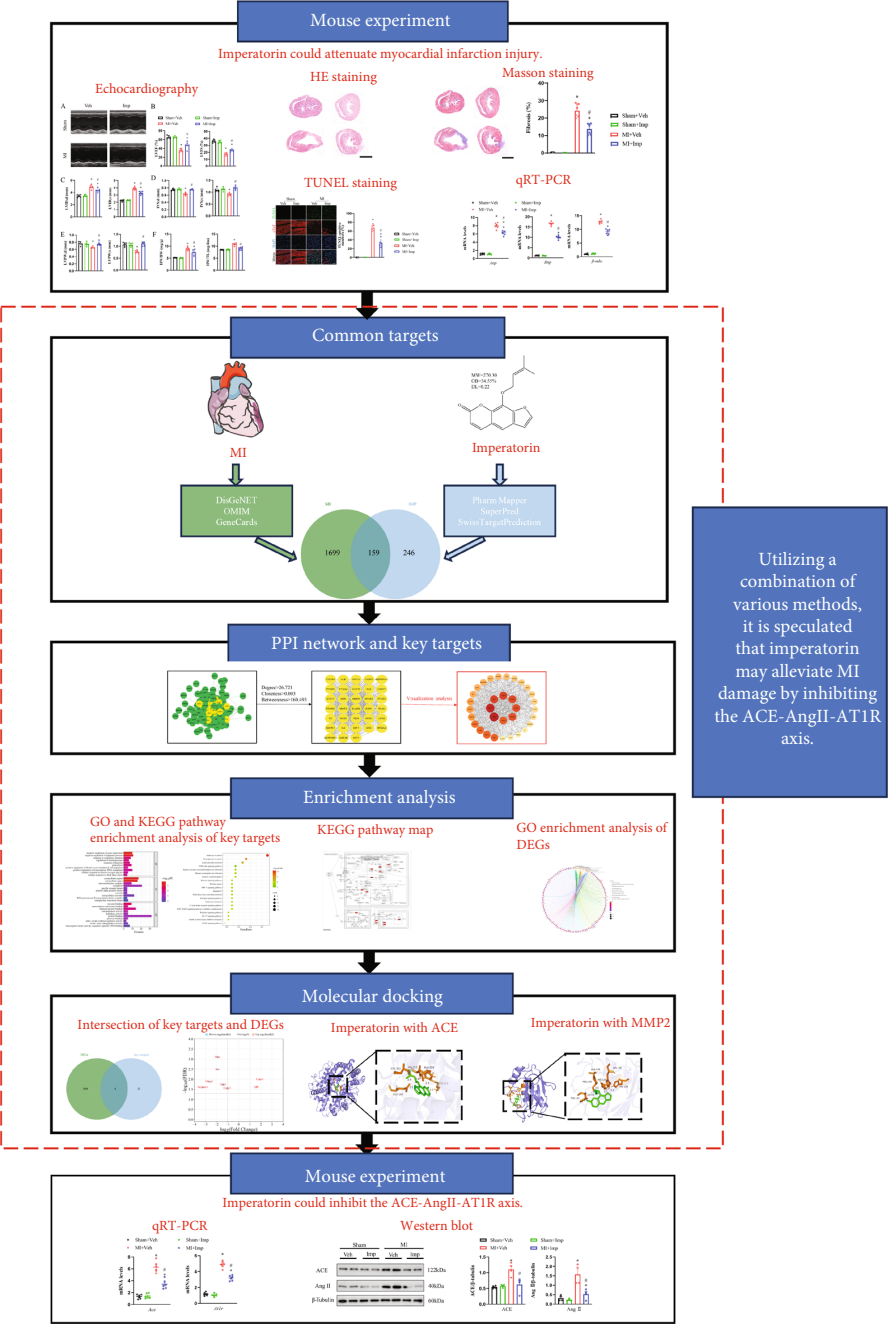
Flowchart.

Recently, advances in network pharmacology improved current understanding of the function of complex bioactive substances in numerous kinds of medicinal plants [13]. Molecular docking allows the discovery of small molecules that dock into the structure of larger molecules, allowing scoring of their complementarity at binding sites [14]. Using an experimental MI mouse model, this study confirmed imperatorin's efficacy before applying network pharmacology for discovering the compound's key targets as well as the core signaling pathways involved. Furthermore, differentially expressed genes (DEGs) were identified between the MI mouse model with and without imperatorin treatment, which were overlapped with key target genes to obtain the target proteins for molecular docking with imperatorin. The results revealed that imperatorin suppressed the ACE–angiotensin II (Ang II)–angiotensin II Type 1 receptor (AT1R) axis. Finally, this hypothesis was confirmed by demonstrating that imperatorin reduced the *Ace* and *At1r* mRNA expression and ACE and Ang II protein expression in MI mice. The flowchart illustrates the entire process (Figure 1).

## 2. Methods

### 2.1. Network Pharmacology and Molecular Docking

#### 2.1.1. Identification of Common Targets

The Traditional Chinese Medicine Systems Pharmacology Database and Analysis Platform (TCMSP) was used to obtain data on the structure, pharmacological, and molecular properties of imperatorin [15]. The Simplified Molecular Input Line Entry System (SMILES) of imperatorin was obtained from PubChem [16]. The druglikeness (DL) and pharmacokinetics of imperatorin were assessed using SwissADME [17]. The above-mentioned chemical structure file was uploaded into the PharmMapper database, SwissTargetPrediction database, and SuperPred database to acquire the potential targets of imperatorin [18–20]. The DisGeNET database, OMIM database, and GeneCards database were used to collect MI-related targets using the search term “myocardial infarction” [21–23]. The intersection method was used to find common targets between MI-related targets and imperatorin predicted targets. A Venn diagram showing common targets was generated from the bioinformatics platform [24, 25].

#### 2.1.2. Protein–Protein Interaction (PPI) Network Construction and Analysis

The PPI network was obtained by importing the common target genes into the STRING database [26]. The screening parameters were set for the species *Homo sapiens* with a minimum required interaction score of medium confidence (0.400), and unbound genes were excluded. Subsequently, PPI data meeting the screening parameters were imported into Cytoscape software that generated PPI network diagrams [27]. A topological analysis was conducted utilizing the CentiScaPe 2.2 plugin tool, and thresholds for betweenness, closeness, and degree were calculated [28], and based on its results, key targets were selected with betweenness ≥ 160.4936, closeness ≥ 0.0032, and degree ≥ 26.7215. A PPI network diagram featuring the identified key targets was generated, with the color and node size adapted according to their degree of connectivity.

#### 2.1.3. Enrichment Analysis of Gene Ontology (GO) Terms and Kyoto Encyclopedia of Genes and Genomes (KEGG) Pathways

For the key targets, GO and KEGG enrichment analysis was performed using the Database for Annotation, Visualization and Integrated Discovery (DAVID) [29], with a false discovery rate (FDR) of < 0.05 and a *p* value of < 0.05 selected as thresholds for considering GO terms and KEGG pathways as statistically significant. Results were eventually visualized on the bioinformatics platform.

#### 2.1.4. Molecular Docking

The proteins derived from the target DEGs were selected for molecular docking. The protein data bank (PDB) format files of key proteins as well as the mol2 format files of imperatorin were acquired from the PDB and the TCMSP, respectively. The molecular docking of small molecule with proteins was performed using AutoDock Tools [30]. The docking outcomes were visualized using PyMOL.

### 2.2. Mice

Forty-eight 8-week-old male C57BL/6 mice were obtained from the Animal Core of Nanjing Medical University and housed in a controlled environment having a light/dark cycle of under 12 h, a temperature of 22 ± 1°C, and a relative humidity of 30%–40%, with food and water also freely accessible to the animals.

### 2.3. Mouse MI Model and Administration Protocol

**Figure 2:**
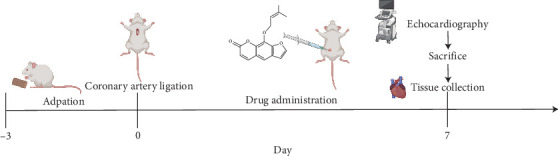
Experimental timeline.

The diagram provides an overview of the experimental timeline (Figure 2). A mouse model of MI was established by inducing permanent left anterior descending ligation in C57BL/6 mice, according to a previous procedure [31]. After anesthesia with isoflurane, the mice were connected to a small animal ventilator for ventilation. Thoracotomy was then performed on the left side of the mouse and was followed by ligation of the left anterior descending coronary artery with a 7-0 nonabsorbable surgical suture. The chest opening and skin were fully sealed using 5-0 stitches. The sham group received the same surgical procedure without the above ligation.

Fifteen milligrams per milliliter imperatorin (Imp, MedChemExpress) clear solution was obtained by sequentially adding each solvent including 1% DMSO, 40% PEG300, 5% Tween-80, and saline. Mice were then randomly assigned to the following groups: sham + Veh group (*n* = 12), sham + Imp group (*n* = 12), MI + Veh group (*n* = 12), and MI + Imp group (*n* = 12). Based on literature and preliminary experiments, a dose of 15 mg/kg/day was determined to be suitable and effective for this study [32, 33]. Imperatorin was intraperitoneally injected into mice once a day for 1 week beginning on the day of the surgery. The vehicle group received an equivalent volume of solvent.

### 2.4. Echocardiography

Echocardiography was performed as previously described [34]. One week after MI operation, mice were reanesthetized with isoflurane. A Vevo 3100 Imaging System (VisualSonics) was then used to obtain the following echocardiographic measurements from unconscious mice: systolic left ventricular posterior wall thickness (LVPW;s), systolic interventricular septal thickness (IVS;s), systolic left ventricular internal diameter (LVID;s), diastolic left ventricular posterior wall thickness (LVPW;d), diastolic interventricular septal thickness (IVS;d), diastolic left ventricular internal diameter (LVID;d), fractional shortening (FS), left ventricular ejection fraction (EF), heart weight to tibia length ratio (HW/TL) and heart weight to body weight ratio (HW/BW).

### 2.5. Histological Analysis

Histological analysis was conducted as previously reported [35]. After removing the hearts of mice, they were paraffinized and cut into sections (4-*μ*m thick). This was followed by Masson's trichrome staining (Servicebio) as well as hematoxylin and eosin staining (Servicebio) for assessing the morphology, while the percentage of fibrosis was determined based on the ratio of the fibrotic area to the total area of the heart. Sections were subsequently examined under a light microscope. The terminal deoxynucleotidyl transferase dUTP nick-end labeling (TUNEL) BrightGreen Apoptosis Detection Kit (Vazyme), cTnT (Proteintech), and DAPI (Beyotime) were then used to stain myocardial tissue sections, cardiomyocytes, and nuclei, respectively, before visualization and image capture with a confocal microscope. The assessment of fibrosis and the percentage of TUNEL-positive cells were evaluated using ImageJ software.

### 2.6. RNA Sequencing and GO Analysis

After extracting RNA from the collected hearts using Trizol (R401-01, Vazyme, China), the samples were sent to Frasergen Genomic Medicine (Wuhan, China) to assess RNA concentration and purity with a spectrophotometer (NanoDrop One, Thermo) as well as RNA integrity with an Agilent Bioanalyzer instrument (Agilent Technologies). Differential analysis was conducted with criteria of FDR < 0.05, *p* value < 0.05, and a fold change of > 2 or < 0.5. A GO analysis was performed to detect the enriched molecular functions (MFs).

### 2.7. Quantitative Reverse Transcriptase Polymerase Chain Reaction (qRT-PCR)

Following RNA extraction from heart samples using Trizol, a corresponding cDNA strand was synthesized with RT SuperMix (Vazyme) prior to qRT-PCR using an SYBR Green Mix (Q131-02, Vazyme, China).  Table 1 shows the primers used for the amplification step. The 2^−*ΔΔ*ct^ method was eventually used to determine relative gene expression, with *α*-actinin acting as the reference.

### 2.8. Western Blotting

Heart samples were subjected to sonication in ice-cold RIPA buffer (P0013B, Beyotime, China) to extract total proteins, with the concentration subsequently measured using a BCA protein concentration assay kit (20201ES76, Yeasen, China). Conventional sodium dodecyl sulfate-polyacrylamide gel electrophoresis (SDS-PAGE) separation was then performed, followed by membrane transfer, membrane blocking using 5% skim milk, and incubation with the primary antibodies including anti-*β*-tubulin (1:1000, Santa Cruz Biotechnology), anti-Ang II (1:1000, Abcam), and anti-ACE (1:1,000, Abcam). After washing the membrane, a second hybridization was performed with secondary antibodies, and chemiluminescent detection kit was used to visualize the gray bands. Protein band grayscale was quantified using ImageJ software.

### 2.9. Statistics Analysis

Statistical analyses were carried out with GraphPad Prism 10, and the results were expressed as means ± standard error of the mean (SEM). One-way analysis of variance (ANOVA) and *t*-tests were then performed for multiple-group and two-group comparisons, respectively, with statistical significance considered at *p* < 0.05.

## 3. Results

### 3.1. Feasibility of Imperatorin as a Drug

Oral bioavailability (OB) refers to the proportion of an orally administered dose of a drug that reaches systemic circulation unchanged, while DL assesses the extent to which a potential compound exhibits the typical characteristics of a drug [36]. A previous research demonstrated that compounds for which the OB is ≥ 30% possess an increased oral absorption but reduced metabolism [37]. Similarly, compounds for which the DL is ≥ 0.18 possess an enhanced effectiveness, being feasible for drug development [38]. The pharmacokinetic parameters of imperatorin (molecular weight = 270.30, OB = 34.55%, DL = 0.22, and blood–brain barrier = 0.92), obtained from TCMSP indicated good absorption, distribution, metabolism, and excretion (ADME) properties and biological activity. Additionally, SwissADME shows imperatorin complies with the Lipinski et al. [39]; Ghose, Viswanadhan, and Wendoloski [40]; Veber et al. [41]; Egan, Merz, and Baldwin [42]; and Muegge, Heald, and Brittelli drug-like rules [43], indicating its promising drug potential. The results show a “high” rating for gastrointestinal absorption, suggesting that imperatorin has good OB. The various indications mentioned above suggested the potential effect of imperatorin on MI.

### 3.2. Validation of the Efficacy of Imperatorin on MI

**Figure 3:**
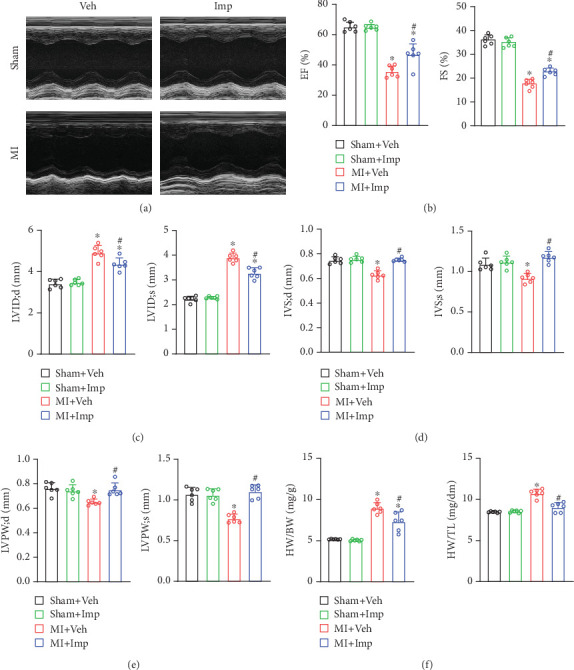
Imperatorin attenuated myocardial infarction (MI) injury as revealed by echocardiographic measurements. Mice were intraperitoneally treated with vehicle (Veh) or imperatorin (Imp) for 1 week after the induction of myocardial infarction. (a) Representative M-mode echocardiogram. (b) Echocardiographic measurements of the left ventricular ejection fraction (EF, %) and fractional shortening (FS, %). (c) Echocardiographic measurements of diastolic left ventricular internal diameter (LVID;d, millimeter) and systolic left ventricular internal diameter (LVID;s, millimeter). (d) Echocardiographic measurements of diastolic interventricular septal thickness (IVS;d, millimeter) and systolic interventricular septal thickness (IVS;s, mm). (e) Echocardiographic measurements of diastolic left ventricular posterior wall thickness (LVPW;d, millimeter), systolic left ventricular posterior wall thickness (LVPW;s, millimeter). (f) Echocardiographic measurements of heart weight to body weight ratio (HW/BW, milligram per gram) and heart weight to tibia length ratio (HW/TL, milligram per decimeter). Results are presented as mean ± SEM. ⁣^∗^*p* < 0.05 versus sham, ^#^*p* < 0.05 versus MI. *n* = 6.

Mice were subjected to MI surgery and subsequent treatment with imperatorin to determine its efficacy on MI.  Table 2 shows echocardiographic results of each group of mice. The representative echocardiogram demonstrated left ventricular wall thinning in MI mice, while the administration of imperatorin mitigated cardiac injury (Figure 3(a)). Imperatorin treatment resulted in an enhancement in EF, which is a crucial indicator of cardiac function, when compared to the vehicle treatment in infarcted hearts, and a significant improvement was also observed in FS (Figure 3(b)). Furthermore, the parameters of cardiac structure such as LVPW;s, IVS;s, LVID;s, LVPW;d, IVS;d, and LVID;d were markedly improved by imperatorin in MI mice, s Indicating improved cardiac function in infarcted hearts (*p* < 0.05 for all) (Figures 3(c), 3(d), and 3(e)). Imperatorin reduced the MI-induced increases in HW/TL and HW/BW, indicating that it can improve ventricular remodeling following MI (*p* < 0.05 for both) (Figure 3(f)).

**Figure 4:**
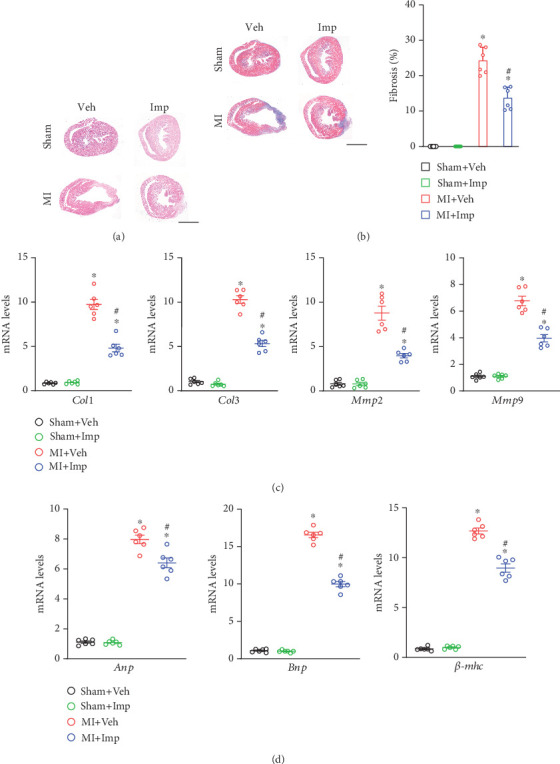
Imperatorin attenuated myocardial infarction–induced fibrosis and heart failure. (a) Representative images of hematoxylin and eosin staining (scale bar: 1 mm). (b) Representative images of Masson trichrome staining (scale bar: 1 mm) and quantification of the positive cells. (c) mRNA expression of fibrosis-related genes *Col1*, *Col3*, *Mmp2*, and *Mmp9*. (d) mRNA expression of the heart failure markers *Anp*, *Bnp*, and *β-mhc*. Results are presented as mean ± SEM. ⁣^∗^*p* < 0.05 versus sham, ^#^*p* < 0.05 versus MI. *n* = 6.

The hearts of sham mice showed normal morphology, with no signs of necrosis or architectural loss. In contrast, cardiac fibrosis was evident in MI mice (Figure 4(a)). The percentage of cardiac fibrosis was significantly reduced with imperatorin treatment compared to the MI group (*p* < 0.05) (Figure 4(b)). qRT-PCR results indicated that MI significantly increased the mRNA levels of fibrosis-related genes (*Col1*, *Col3*, *Mmp2*, and *Mmp9*) and heart failure biomarkers (*Anp*, *Bnp*, and *β-Mhc*) compared to the sham group (*p* < 0.05 for all). However, post-MI treatment with imperatorin significantly decreased the expression levels of these genes (*p* < 0.05 for all) (Figures 4(c) and 4(d)). These findings suggested that imperatorin may help prevent adverse cardiac fibrosis and improve heart function following MI.

**Figure 5:**
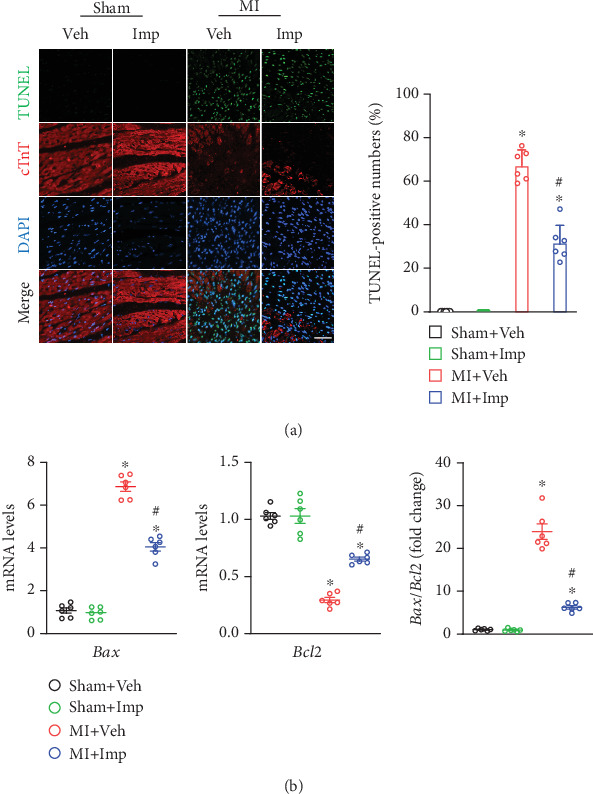
Imperatorin reduced myocardial cell apoptosis in infarcted mice. (a) Representative images of TUNEL staining (scale bar: 50 *μ*m) and quantification of the positive cells. (b) mRNA expression of the level ratio of *Bax/Bcl2*. Results are presented as mean ± SEM. ⁣^∗^*p* < 0.05 versus sham, ^#^*p* < 0.05 versus MI. *n* = 6.

The myocardial sections of the MI + Imp group (31.85% ± 8.34%) had a significantly lower number of TUNEL-positive cells compared to the MI + Veh group (67.5% ± 6.65%) (*p* < 0.05) (Figure 5(a)). Moreover, while the *Bcl2/Bax* mRNA ratio was reduced in infarcted hearts, this reduction was prevented by imperatorin treatment (*p* < 0.05) (Figure 5(b)). These findings indicated that imperatorin reduced cardiomyocyte apoptosis in infarcted hearts.

### 3.3. Targets for Imperatorin in MI and PPI Network Construction and Analysis

**Figure 6:**
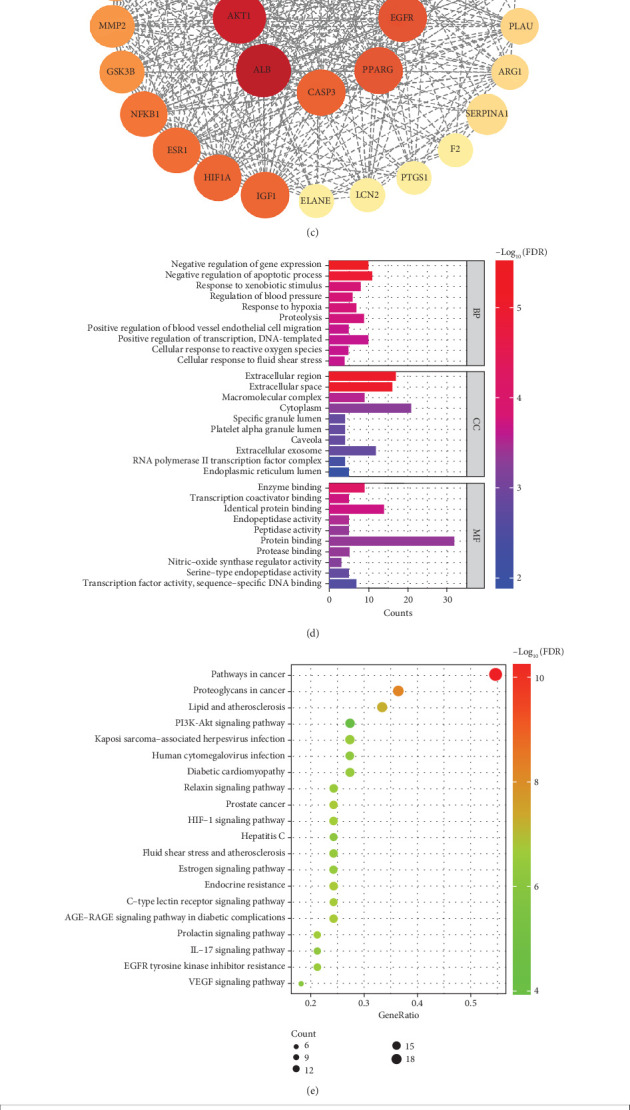
Targets for imperatorin in by GO and KEGG pathway enrichment analysis. (a) Common targets between imperatorin and myocardial infarction shown by the Venn diagram and protein–protein interaction (PPI) network, generated by the STRING database. (b) Key target genes were chosen based on criteria such as betweenness ≥ 160.4936, closeness ≥ 0.0032, and degree ≥ 26.7215, identified through common target genes. (c) PPI network of key targets. The size of the points increases from small to large, and the color deepens from light to dark, representing the increase in degree from small to large. (d) GO enrichment analysis (ranking by FDR). FDR, false discovery rate. BP, biological process. CC, cellular component. MF, molecular function. The *x*-axis represents the gene number in each category. The color gradient represents various FDR thresholds. (e) KEGG pathway enrichment analysis (ranking by gene ratio). The *x*-axis indicates the gene ratio, which is defined as the ratio of the number of genes in a specific gene list to the total number of genes involved in a particular pathway. The *y*-axis indicates the enriched pathway name. The color gradient represents various FDR thresholds, and the dot size corresponds to the number of genes associated with each pathway. (f) KEGG pathway map of diabetic cardiomyopathy. Red represents the genes related to pathways within key targets.

Overall, 405 targets of imperatorin were predicted and 1858 targets related to MI were identified. Their intersection identified 159 common targets. The Venn diagram was created, and the PPI network was constructed (Figure 6(a)) to get 33 key targets (Figure 6(b)). A network diagram of 33 key targets was generated, with the color and node size adjusted according to their degree of connectivity (Figure 6(c)).

### 3.4. Enrichment Analysis of GO Terms and KEGG Pathways

The DAVID database was used to perform GO and KEGG pathway enrichment analysis on the 33 key targets of imperatorin treatment for MI.

GO analysis identified 121 GO functional enrichment entries of which 18, 78, and 25 were linked to cellular component (CC), biological process (BP), and MF, respectively. The categories related to BP, CC, and MF were arranged in an ascending order of FDR values, with the top 10 entries selected for each category, and the results (Figure 6(d)) revealed that BP mainly included entries of regulation of blood pressure and response to hypoxia; CC mainly included the cytoplasm and extracellular region, and MF mainly included transcription coactivator binding and endopeptidase activity. These entries involved gene expression, cellular physiological activities, blood pressure regulation, stress response, and other aspects, which may be closely associated with the onset and progression of MI.

In addition, 110 KEGG signaling pathways were obtained. The top 20 were arranged in an ascending order of FDR value (Figure 6(e)), indicating the involvement of the targets associated with several processes, including lipid and atherosclerosis, diabetic cardiomyopathy, and PI3K-Akt signaling pathway. Additionally, imperatorin was highly associated with diabetic cardiomyopathy induced by MI among these enriched pathways. The predicted targets corresponding to this signaling pathway revealed that the ACE–Ang II–AT1R axis was involved (Figure 6(f)).

### 3.5. RNA Sequencing and GO Analysis

**Figure 7:**
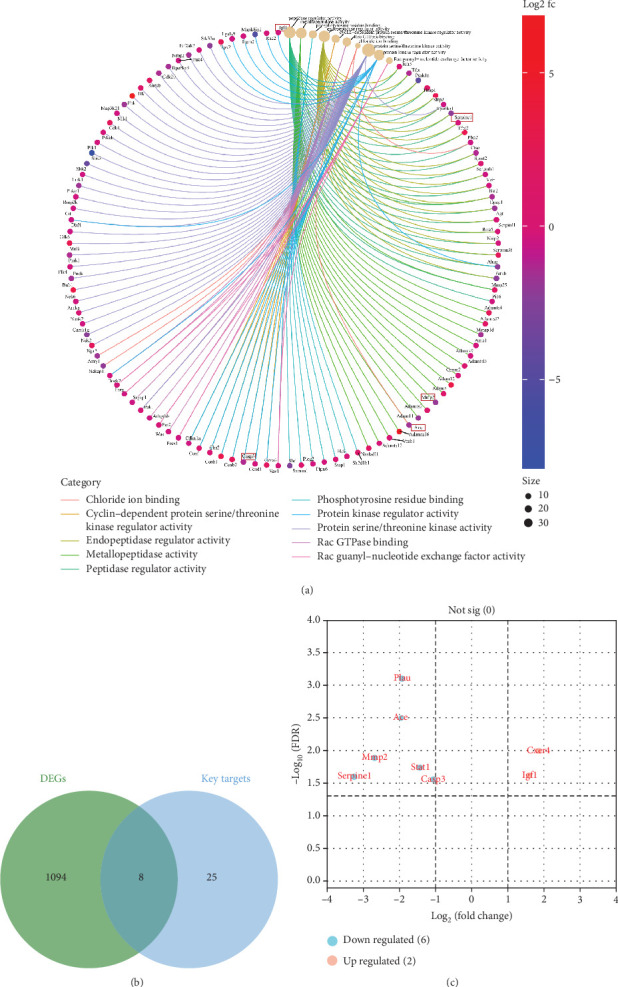
Functional enrichment based on differentially expressed genes (DEGs) between the myocardial infarction model mice with and without imperatorin, acquisition of common genes of DEGs and key targets, and volcano plot of key targets. (a) GO–MF analysis for DEGs showing the significant terms by a cnetplot. The color gradient represents various fold change thresholds, and the dot size corresponds to the number associated with each pathway. The key target genes differentially expressed in mice are listed in the box including *Ace*, *Mmp2*, *Serpine1*, *Igf1*, and *Casp3*. (b) Common genes between DEGs and key targets shown by the Venn diagram. (c) Volcano plot showing the distribution of common genes between DEGs and key targets. Red, blue, and gray dots represent gene expression corresponding to upregulated, downregulated, and not significant expression, respectively.

RNA sequencing was performed on cardiac tissues from mice in the MI + Imp and MI groups to identify gene expression changes induced by imperatorin administration. A total of 1102 DEGs were identified from the RNA sequencing data. The top 10 GO–MF-enriched pathways indicated that the 1102 DEGs were associated with various activities, including peptidase regulator activity, metallopeptidase activity, phosphotyrosine residue binding, endopeptidase regulator activity, Rac GTPase binding, and chloride ion binding (Figure 7(a)). Among these DEGs, eight genes overlapped with the 33 predicted therapeutic targets, including *Casp3*, *Igf1*, *Mmp2*, *Cxcr4*, *Serpine1*, *Stat1*, *Ace*, and *Plau* (Figure 7(b)). Of these, two genes were upregulated, and six were downregulated (Figure 7(c)). Notably, *Mmp2* was enriched in metallopeptidase activity, while *Ace* was enriched in both metallopeptidase activity and chloride ion binding.

### 3.6. Molecular Docking Analysis

**Figure 8:**
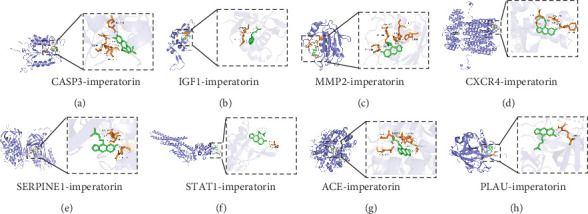
Molecular docking diagram showing the binding interactions of imperatorin with eight key target proteins. (a) Caspase-3 (CASP3)-imperatorin, (b) insulin-like growth factor 1 (IGF1)-imperatorin, (c) matrix metalloproteinase-2 (MMP2)-imperatorin, (d) C-X-C chemokine receptor type 4 (CXCR4)-imperatorin, (e) plasminogen activator inhibitor-1 (SERPINE1)-imperatorin, (f) signal transducer and activator of transcription 1 (STAT1)-imperatorin, (g) angiotensin-converting enzyme (ACE)-imperatorin, (h) urokinase-type plasminogen activator (PLAU)-imperatorin. The red dashed lines indicate the hydrogen bonds, with the interaction distances labeled above the lines. The green sticks represent imperatorin, and the yellow sticks represent amino acid residues.

Proteins encoded by the eight overlapping genes were selected for molecular docking with imperatorin. A molecular docking diagram shows the binding site simulation (Figure 8). Binding potential is indicated by a binding energy of less than −5 kcal/mol for binding, while a stronger binding affinity between two entities is indicated by a value less of than −7 kcal/mol [44].  Table 3 shows the docking results of key target proteins with imperatorin molecules. In the case of imperatorin and the target proteins, the interaction affinity was found to be consistently below −5 kcal/mol, except for STAT1. The formation of hydrogen bonds between imperatorin and target proteins stabilized small molecules, also suggesting that imperatorin matched the active pocket of the target proteins. Of the eight proteins, imperatorin formed the greatest number of hydrogen bonds with MMP2, all with the lowest binding energy, indicating the tightest binding between imperatorin and MMP2. Similarly, imperatorin formed hydrogen bonds with ACE at GLU-162 (2.5 Å), CYS-352 (3.4 Å), HIS-353 (2.4 Å), ALA-354 (2.3 Å), and LYS-511 (2.0 Å), with relatively shorter average bond lengths and stronger binding affinity, indicating a tight and robust binding ability between them.

### 3.7. Mechanism of Action of Imperatorin on MI

Notably, ACE and MMP2 proteins had a higher number of hydrogen bonds and stronger binding affinity with imperatorin. In the top 10 enriched pathways of DEGs in the GO–MF category, ACE was enriched in chloride ion binding, which may be associated with reducing the risk of MI. Thus, ACE was the most critical target in the mechanism of action of imperatorin in treating MI. Among the top 10 KEGG-enriched pathways of the 33 key targets, diabetic cardiomyopathy showed a significant correlation with the effectiveness of imperatorin in treating MI, and the ACE–Ang II–AT1R axis resulted involved in this disease. Therefore, the target of the ACE–Ang II–AT1R axis might be a promising approach for attenuating myocardial injury and improving the outcomes in MI patients. Our hypothesis was that imperatorin blocked the ACE–Ang II–AT1R axis.

### 3.8. Imperatorin Inhibited the ACE–Ang II–AT1R Axis Involved in MI In Vivo

**Figure 9:**
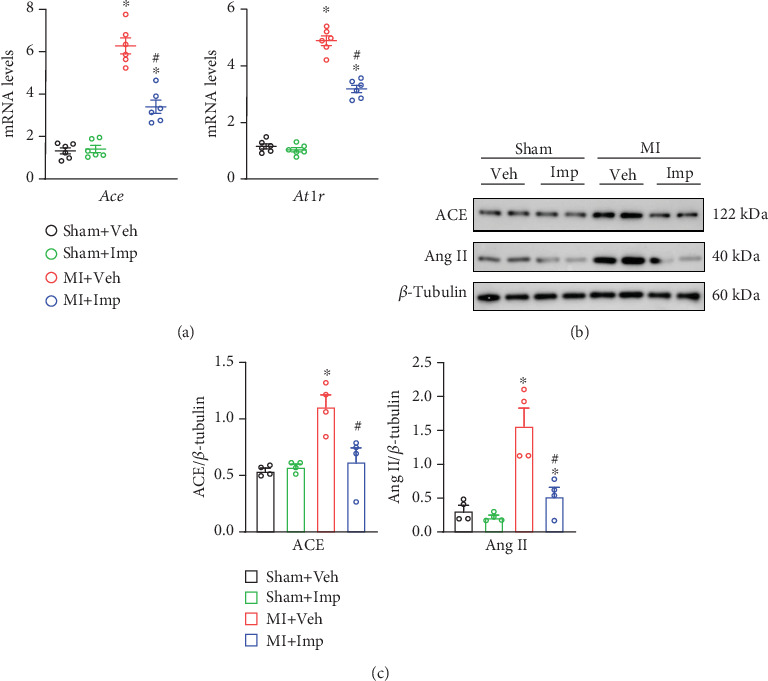
Imperatorin inhibits ACE-Ang II–AT1R axis. (a) *Ace*, *At1r* mRNA expression by qRT-PCR (*n* = 6 in each group). (b) ACE and Ang II detected by western blot. (c) Quantification of ACE and Ang II protein expression (*n* = 4 in each group). Results are presented as mean ± SEM. ⁣^∗^*p* < 0.05 versus sham, ^#^*p* < 0.05 versus MI.

Our in vivo experiments supported the hypothesis by demonstrating that the mRNA expression levels of *Ace* and *At1r* were significantly elevated in the MI group compared to the sham group (*p* < 0.05 for both) (Figure 9(a)). However, treatment with imperatorin markedly reduced the expression of both genes (*p* < 0.05 for both). The protein analysis results were consistent with these findings. ACE and Ang II protein levels were significantly higher in the MI group compared to the sham group (*p* < 0.05 for both) (Figure 9(b)), but imperatorin treatment led to a significant reduction in both protein levels (*p* < 0.05 for both), mirroring the mRNA expression patterns. The original uncropped western blot image corresponding to Figure 9(b) is provided in the Supporting Information (Figure S1). These results highlight imperatorin's potential role in reducing both mRNA and protein expressions related to MI.

## 4. Discussion

**Figure 10:**
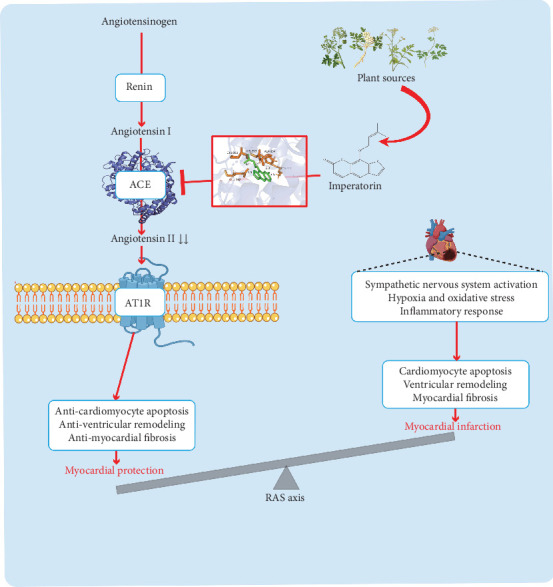
Mechanism diagram of imperatorin in treating myocardial infarction. Red standard arrows indicate activating effects, while red T-shaped arrows represent inhibitory effects (adapted from “Brain Renin Angiotensin System”, by BioRender.com. Retrieved from https://app.biorender.com/biorender-templates).

The diagram illustrates the mechanism by which imperatorin treats MI (Figure 10). During MI, ischemia and injury trigger a series of molecular and cellular responses, notably the activation of the renin–angiotensin system (RAS). The interruption of blood flow causes myocardial cells to become hypoxic, leading to oxidative stress and the production of reactive oxygen species, which directly damage cells and activate RAS through various signaling pathways [45, 46]. MI is also accompanied by a robust inflammatory response [47]. Ischemic injury prompts damaged myocardial cells to release numerous inflammatory mediators and cytokines, which not only attract immune cells, such as neutrophils and macrophages, to the injury site but also directly or indirectly activate RAS [48]. Additionally, the activation of the sympathetic nervous system is a response to acute stress following MI, increasing heart rate and myocardial contractility through the release of norepinephrine and epinephrine [49]. These catecholamines not only enhance cardiac output but also stimulate renin release, thereby initiating and amplifying the RAS pathway [50]. In summary, MI activates the RAS through multiple pathways, including hypoxia and oxidative stress, inflammation, and sympathetic nervous system activation.

Activation of the RAS elevates Ang II levels, which in turn activate signaling pathways like p38 MAPK and JNK, ultimately leading to cardiomyocyte apoptosis [51]. Additionally, Ang II induces cardiomyocyte hypertrophy, fibroblast proliferation, and increased collagen synthesis, collectively altering the structure and function of the ventricular wall and resulting in ventricular remodeling [52, 53]. Furthermore, Ang II activates the TGF-*β* signaling pathway, promoting fibroblast differentiation into myofibroblasts and increasing collagen deposition, which leads to myocardial fibrosis [54, 55]. These pathological processes contribute significantly to the progression of MI. Therefore, pharmacological interventions targeting RAS, such as ACE inhibitors and angiotensin II receptor blockers (ARBs), are of significant clinical importance in the treatment of MI [56, 57].

Our hypothesis is that imperatorin exerts its cardioprotective effects by blocking the ACE–Ang II–AT1R axis. Animal experiments demonstrated significant improvements in cardiac function and structure in MI mice following treatment with imperatorin. It mitigated left ventricular dilation, preserved cardiac function, attenuated cardiac remodeling and fibrosis, and reduced the number of apoptotic cardiomyocytes, indicating its potential protective effect against myocardial injury induced by MI. In vivo experiments demonstrated a significant reduction in *Ace* and *At1r* mRNA expression as well as in ACE and Ang II protein expression in cardiac tissues after imperatorin treatment compared with the MI group. The results suggest that imperatorin reduced MI damage by inhibiting the ACE–Ang II–AT1R axis.

**Figure 11:**
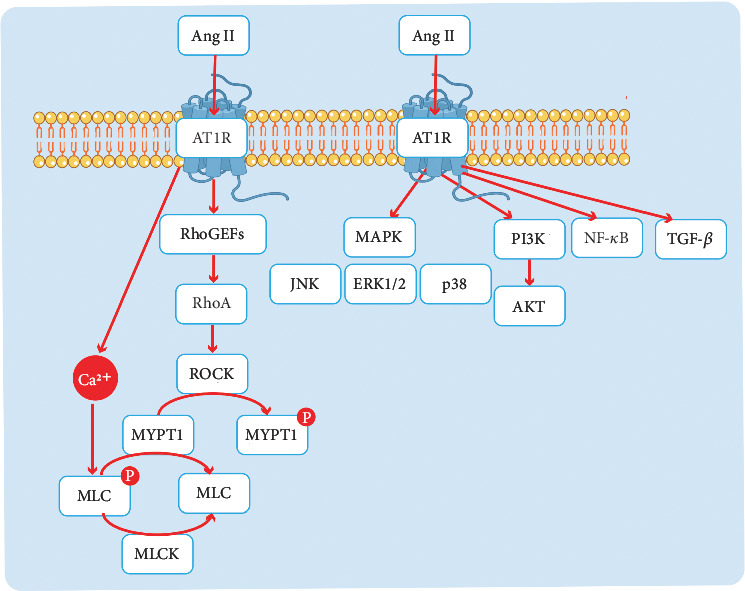
The downstream components of the ACE–Ang II–AT1R axis. Red standard arrows indicate activating effects. RhoGEFs, Rho guanine nucleotide exchange factors. RhoA, Ras homolog family member A. ROCK, Rho-associated kinase. MYPT1, myosin phosphatase target subunit 1. MLC, myosin light chain. MLCK, myosin light chain. MAPK, mitogen-activated protein kinase. ERK1/2, extracellular signal-regulated kinase 1/2. JNK, c-Jun n-terminal kinase. PI3K, phosphoinositide 3-kinase. Akt, protein kinase B. NF-*κ*B, nuclear factor kappa-light-chain-enhancer of activated B cells. TGF-*β*, transforming growth factor beta.

The mechanism diagram illustrates how activation of the ACE–Ang II–AT1R signaling pathway triggers multiple downstream cascades that contribute to cardiovascular pathophysiology (Figure 11). A key pathway involved is the MAPK pathway, which includes ERK1/2, JNK, and p38, and plays a critical role in cellular responses such as inflammation and hypertrophy [58–61]. The PI3K-Akt pathway is also activated, leading to cell survival and migration [62, 63], while NF-*κ*B activation contributes to inflammatory and immune response [64, 65]. Furthermore, the TGF-*β* pathway is implicated in vascular remodeling and fibrosis [66, 67]. In recent years, research on the ACE–Ang II–AT1R signaling pathway has begun to focus on the Ras homolog family member A (RhoA) pathway, a Ca^2+^-sensitizing mechanism that facilitates vascular smooth muscle contraction even at low intracellular Ca^2+^ levels [68, 69]. The activation of AT1R elevates intracellular Ca^2+^ levels and stimulates myosin light chain kinase (MLCK), resulting in myosin light chain (MLC) phosphorylation and smooth muscle contraction [70]. RhoA activity is modulated by Rho guanine nucleotide exchange factors (RhoGEFs), which serve as intermediaries, linking AT1R activation to downstream RhoA signaling [71, 72]. Upon activation by G protein-coupled receptors like AT1R, RhoGEFs promote the exchange of GDP for GTP on RhoA, activating it. Activated RhoA then stimulates Rho-associated kinase (ROCK), which phosphorylates the myosin phosphatase target subunit 1 (MYPT1), inhibiting MYPT1 and thereby increasing MLC phosphorylation, leading to vasoconstriction.

Currently, the studies on imperatorin primarily focus on its antitumor effects, while studies on its cardioprotective effects are limited and mostly performed in hypertension models. No reports are available on the pharmacological and molecular targets of imperatorin on MI. Imperatorin exhibits a wide range of pharmacological activities, but elucidating its mechanisms in treating MI through traditional animal and cell experiments requires significant effort. To address this, we employed network pharmacology and molecular docking as in silico methods, while simultaneously establishing an MI mouse model for in vivo studies. By combining these two approaches, we thoroughly revealed the therapeutic effects and mechanisms of imperatorin on MI, providing a crucial theoretical foundation for its future clinical application in treating this condition. In addition, our research also provides an approach for exploring the mechanisms by which traditional Chinese medicine treats diseases.

Nevertheless, certain limitations are present in this study. First, we focused on one signaling pathway, but MI is a complex disease involving multiple BPs. Therefore, examining additional pathways is essential for a comprehensive understanding of the drug's mechanism of action. Further assessment of more signaling pathways should be performed for comprehensively understanding imperatorin's mechanism of action. Second, our in vivo experiment was limited to an MI mouse model, so additional experiments, including clinical research, are required for confirming the safety and efficacy of imperatorin as a therapeutic agent for MI.

## 5. Conclusion

Our study demonstrated the efficacy of imperatorin on MI model mice, with its inhibitory effect on the ACE–Ang II–AT1R axis playing a crucial role in MI.

## Figures and Tables

**Table 1:** List of primer sequences used in qRT-PCR analysis.

**Names**	**Forward primer (5**⁣′**-3**⁣′**)**	**Reverse primer (5**⁣′**-3**⁣′**)**
*Col1*	TGGCATCTTCTCCTTCCAGC	ACGTCCTGGCAGCCATGTC
*Col3*	ACGTAGATGAATTGGGATGCAG	GGGTTGGGGCAGTCTAGTG
*Mmp2*	CCCGATCTACACCTACACCAA	AAACCGGTCCTTGAAGAAGAA
*Mmp9*	CCCGATCTACACCTACACCAA	AAGATGAACGGGAACACACAG
*Anp*	ACCTCCCGAAGCTACCTAAGT	CAACCTTTTCAACGGCTCCAA
*Bnp*	GAGGTCACTCCTATCCTCTGG	GCCATTTCCCTCCGACTTTTC
*β-mhc*	ATGTGCCGGACCTTGGAA	CCTCGGGTTAGCTGAGAGATCA
*Bcl2*	TACCGTCGTGACTTCGCAGAG	GGCAGGCTGAGCAGGGTCTT
*Bax*	AGACAGGGGCCTTTTTGCTAC	AATTCGCCGGAGACACTCG
*Ace*	TTCACATCCCAAGCGTGACA	CTGAACCCACCAGGTCCTTC
*At1r*	GGGCTGTCTACACTGCTATGGAA	CCGGAAGCGATCTTACATAGGTA
*α-Actin*	CAGCTGAGAGGGAAATCGTG	CGTTGCCAATAGTGATGACC

**Table 2:** Echocardiographic results of each group of mice (means ± SEM).

**Parameters**	**S** **h** **a** **m** + **Veh**	**S** **h** **a** **m** + **Imp**	**M** **I** + **Veh**	**M** **I** + **Imp**
EF (%)	65.25 ± 3.11	64.85 ± 1.99	35.80 ± 3.51	46.86 ± 6.78
FS (%)	36.07 ± 2.07	34.93 ± 1.99	17.58 ± 1.89	22.69 ± 1.56
LVID;d (mm)	3.42 ± 0.21	3.48 ± 0.14	4.92 ± 0.34	4.36 ± 0.30
LVID;s (mm)	2.22 ± 0.12	2.28 ± 0.05	3.90 ± 0.19	3.28 ± 0.22
IVS;d (mm)	0.75 ± 0.03	0.76 ± 0.02	0.62 ± 0.03	0.75 ± 0.01
IVS;s (mm)	1.09 ± 0.08	1.12 ± 0.07	0.92 ± 0.06	1.18 ± 0.07
LVPW;d (mm)	0.76 ± 0.05	0.74 ± 0.05	0.65 ± 0.02	0.75 ± 0.06
LVPW;s (mm)	1.07 ± 0.08	1.06 ± 0.08	0.77 ± 0.05	1.10 ± 0.08
HW/BW (mg/g)	5.17 ± 0.04	5.04 ± 0.12	8.96 ± 0.67	7.33 ± 1.16
HW/TL (mg/dm)	8.47 ± 0.08	8.53 ± 0.15	10.75 ± 0.50	9.06 ± 0.57

**Table 3:** Docking results of key target proteins with imperatorin molecules.

**Gene symbol**	**Degree**	**Betweenness**	**Closeness**	**PDB**	**Hydrogen bonds**	**Affinity (kcal/mol)**
CASP3	77	356.2952	0.0041	6CL0	5	−6.59
IGF1	76	500.7076	0.0041	1WQJ	2	−8.03
MMP2	62	195.2696	0.0039	3AYU	8	−7.88
CXCR4	61	573.3754	0.0038	3ODU	5	−5.53
SERPINE1	53	416.7992	0.0037	7AQF	3	−7.18
STAT1	52	498.3764	0.0037	7NUF	1	−4.28
ACE	49	586.2912	0.0037	7Q27	5	−7.14
PLAU	37	166.9625	0.0035	7NUF	1	−6.57

## Data Availability

The data and materials supporting the findings of this study are available upon request. Please contact Pingxi Xiao at xpx@njmu.edu.cn for access to the data and materials used in this research.

## Supporting Information

Supporting Information Additional supporting information can be found online in the Supporting Information section. The supporting information includes the original uncropped western blot image corresponding to Figure 9(b) in the main manuscript. This is provided as Figure S1 to ensure the transparency and reproducibility of the experimental data.

## References

[1] Thygesen K., Alpert J. S., Jaffe A. S. (2018). Fourth universal definition of myocardial infarction (2018). *Journal of the American College of Cardiology*.

[2] Damluji A. A., van Diepen S., Katz J. N. (2021). Mechanical complications of acute myocardial infarction: a scientific statement from the American Heart Association. *Circulation*.

[3] (2020). Global burden of 369 diseases and injuries in 204 countries and territories, 1990-2019: a systematic analysis for the Global Burden of Disease Study 2019. *Lancet*.

[4] Roth G. A., Mensah G. A., Johnson C. O. (2020). Global burden of cardiovascular diseases and risk factors, 1990–2019. *Journal of the American College of Cardiology*.

[5] Ibanez B., James S., Agewall S. (2018). 2017 ESC guidelines for the management of acute myocardial infarction in patients presenting with ST-segment elevation: the task force for the management of acute myocardial infarction in patients presenting with ST-segment elevation of the European Society of Cardiology (ESC). *European Heart Journal*.

[6] O’Gara P. T., Kushner F. G., Ascheim D. D. (2013). 2013 ACCF/AHA guideline for the management of ST-elevation myocardial infarction: executive summary: a report of the American College of Cardiology Foundation/American Heart Association Task Force on Practice Guidelines. *Circulation*.

[7] Zhang L., Song J., Kong L. (2020). The strategies and techniques of drug discovery from natural products. *Pharmacology & Therapeutics*.

[8] Deng M., Xie L., Zhong L., Liao Y., Liu L., Li X. (2020). Imperatorin: a review of its pharmacology, toxicity and pharmacokinetics. *European Journal of Pharmacology*.

[9] Nasser M. I., Zhu S., Hu H., Huang H., Guo M., Zhu P. (2019). Effects of imperatorin in the cardiovascular system and cancer. *Biomedicine & Pharmacotherapy*.

[10] Zhang Y., Cao Y., Zhan Y., Duan H., He L. (2010). Furanocoumarins-imperatorin inhibits myocardial hypertrophy both in vitro and in vivo. *Fitoterapia*.

[11] Zhang Y., Wang Q. L., Zhan Y. Z., Duan H. J., Cao Y. J., He L. C. (2010). Role of store-operated calcium entry in imperatorin-induced vasodilatation of rat small mesenteric artery. *European Journal of Pharmacology*.

[12] Cao Y. J., He X., Wang N., He L. C. (2013). Effects of imperatorin, the active component from Radix Angelicae (Baizhi), on the blood pressure and oxidative stress in 2K,1C hypertensive rats. *Phytomedicine*.

[13] Noor F., Tahir Ul Qamar M., Ashfaq U. A., Albutti A., Alwashmi A. S. S., Aljasir M. A. (2022). Network pharmacology approach for medicinal plants: review and assessment. *Pharmaceuticals*.

[14] Saikia S., Bordoloi M. (2019). Molecular docking: challenges, advances and its use in drug discovery perspective. *Current Drug Targets*.

[15] Ru J., Li P., Wang J. (2014). TCMSP: a database of systems pharmacology for drug discovery from herbal medicines. *Journal of Cheminformatics*.

[16] Kim S., Chen J., Cheng T. (2023). PubChem 2023 update. *Nucleic Acids Research*.

[17] Daina A., Michielin O., Zoete V. (2017). SwissADME: a free web tool to evaluate pharmacokinetics, drug-likeness and medicinal chemistry friendliness of small molecules. *Scientific Reports*.

[18] Wang X., Shen Y., Wang S. (2017). PharmMapper 2017 update: a web server for potential drug target identification with a comprehensive target pharmacophore database. *Nucleic Acids Research*.

[19] Daina A., Michielin O., Zoete V. (2019). SwissTargetPrediction: updated data and new features for efficient prediction of protein targets of small molecules. *Nucleic Acids Research*.

[20] Nickel J., Gohlke B. O., Erehman J. (2014). SuperPred: update on drug classification and target prediction. *Nucleic Acids Research*.

[21] Piñero J., Saüch J., Sanz F., Furlong L. I. (2021). The DisGeNET cytoscape app: exploring and visualizing disease genomics data. *Biotechnology Journal*.

[22] Amberger J. S., Bocchini C. A., Schiettecatte F., Scott A. F., Hamosh A. (2015). OMIM.org: Online Mendelian Inheritance in Man (OMIM®), an online catalog of human genes and genetic disorders. *Nucleic Acids Research*.

[23] Stelzer G., Rosen N., Plaschkes I. (2016). The GeneCards suite: from gene data mining to disease genome sequence analyses. *Current Protocols in Bioinformatics*.

[24] Bardou P., Mariette J., Escudié F., Djemiel C., Klopp C. (2014). jvenn: an interactive Venn diagram viewer. *BMC Bioinformatics*.

[25] Tang D., Chen M., Huang X. (2023). SRplot: a free online platform for data visualization and graphing. *PLoS One*.

[26] Szklarczyk D., Kirsch R., Koutrouli M. (2023). The STRING database in 2023: protein-protein association networks and functional enrichment analyses for any sequenced genome of interest. *Nucleic Acids Research*.

[27] Shannon P., Markiel A., Ozier O. (2003). Cytoscape: a software environment for integrated models of biomolecular interaction networks. *Genome Research*.

[28] Scardoni G., Petterlini M., Laudanna C. (2009). Analyzing biological network parameters with CentiScaPe. *Bioinformatics*.

[29] Sherman B. T., Hao M., Qiu J. (2022). DAVID: a web server for functional enrichment analysis and functional annotation of gene lists (2021 update). *Nucleic Acids Research*.

[30] Morris G. M., Huey R., Lindstrom W. (2009). AutoDock4 and AutoDockTools4: automated docking with selective receptor flexibility. *Journal of Computational Chemistry*.

[31] Kong C., Lyu D., He C., Li R., Lu Q. (2021). Dioscin elevates lncRNA MANTIS in therapeutic angiogenesis for heart diseases. *Aging Cell*.

[32] Wang X., Lou Y. J., Wang M. X., Shi Y. W., Xu H. X., Kong L. D. (2012). Furocoumarins affect hepatic cytochrome P450 and renal organic ion transporters in mice. *Toxicology Letters*.

[33] Zhang Y., Cao Y., Duan H., Wang H., He L. (2012). Imperatorin prevents cardiac hypertrophy and the transition to heart failure via NO-dependent mechanisms in mice. *Fitoterapia*.

[34] Du L., Chen J., Wu Y. (2021). Long Non-coding RNA N1LR protects against myocardial ischemic/reperfusion injury through regulating the TGF-*β* signaling pathway. *Frontiers in Cardiovascular Medicine*.

[35] Shen T., Lyu D., Zhang M., Shang H., Lu Q. (2022). Dioscin alleviates cardiac dysfunction in acute myocardial infarction via rescuing mitochondrial malfunction. *Frontiers in Cardiovascular Medicine*.

[36] Xu X., Zhang W., Huang C. (2012). A novel chemometric method for the prediction of human oral bioavailability. *International Journal of Molecular Sciences*.

[37] Ahmed S. S., Ramakrishnan V. (2012). Systems biological approach of molecular descriptors connectivity: optimal descriptors for oral bioavailability prediction. *PLoS One*.

[38] Tao W., Xu X., Wang X. (2013). Network pharmacology-based prediction of the active ingredients and potential targets of Chinese herbal Radix Curcumae formula for application to cardiovascular disease. *Journal of Ethnopharmacology*.

[39] Lipinski C. A., Lombardo F., Dominy B. W., Feeney P. J. (2001). Experimental and computational approaches to estimate solubility and permeability in drug discovery and development settings. *Advanced Drug Delivery Reviews*.

[40] Ghose A. K., Viswanadhan V. N., Wendoloski J. J. (1999). A knowledge-based approach in designing combinatorial or medicinal chemistry libraries for drug discovery. 1. A qualitative and quantitative characterization of known drug databases. *Journal of Combinatorial Chemistry*.

[41] Veber D. F., Johnson S. R., Cheng H. Y., Smith B. R., Ward K. W., Kopple K. D. (2002). Molecular properties that influence the oral bioavailability of drug candidates. *Journal of Medicinal Chemistry*.

[42] Egan W. J., Merz K. M., Baldwin J. J. (2000). Prediction of drug absorption using multivariate statistics. *Journal of Medicinal Chemistry*.

[43] Muegge I., Heald S. L., Brittelli D. (2001). Simple selection criteria for drug-like chemical matter. *Journal of Medicinal Chemistry*.

[44] Trott O., Olson A. J. (2010). AutoDock Vina: improving the speed and accuracy of docking with a new scoring function, efficient optimization, and multithreading. *Journal of Computational Chemistry*.

[45] Vanden Hoek T. L., Li C., Shao Z., Schumacker P. T., Becker L. B. (1997). Significant levels of oxidants are generated by isolated cardiomyocytes during ischemia prior to reperfusion. *Journal of Molecular and Cellular Cardiology*.

[46] McFarlane S. I., Kumar A., Sowers J. R. (2003). Mechanisms by which angiotensin-converting enzyme inhibitors prevent diabetes and cardiovascular disease. *The American Journal of Cardiology*.

[47] Frangogiannis N. G. (2014). The inflammatory response in myocardial injury, repair, and remodelling. *Nature Reviews. Cardiology*.

[48] Swirski F. K., Nahrendorf M. (2013). Leukocyte behavior in atherosclerosis, myocardial infarction, and heart failure. *Science*.

[49] Goldstein D. S. (2010). Adrenal responses to stress. *Cellular and Molecular Neurobiology*.

[50] Thayer J. F., Yamamoto S. S., Brosschot J. F. (2010). The relationship of autonomic imbalance, heart rate variability and cardiovascular disease risk factors. *International Journal of Cardiology*.

[51] Whelan R. S., Kaplinskiy V., Kitsis R. N. (2010). Cell death in the pathogenesis of heart disease: mechanisms and significance. *Annual Review of Physiology*.

[52] Cohn J. N., Ferrari R., Sharpe N. (2000). Cardiac remodeling--concepts and clinical implications: a consensus paper from an international forum on cardiac remodeling. Behalf of an International Forum on Cardiac Remodeling. *Journal of the American College of Cardiology*.

[53] Janicki J. S., Brower G. L. (2002). The role of myocardial fibrillar collagen in ventricular remodeling and function. *Journal of Cardiac Failure*.

[54] Rosenkranz S. (2004). TGF-*β*_1_ and angiotensin networking in cardiac remodeling. *Cardiovascular Research*.

[55] Weber K. T. (1997). Extracellular matrix remodeling in heart failure. *Circulation*.

[56] Pfeffer M. A., Braunwald E., Moyé L. A. (1992). Effect of captopril on mortality and morbidity in patients with left ventricular dysfunction after myocardial infarction. Results of the survival and ventricular enlargement trial. The SAVE Investigators. *The New England Journal of Medicine*.

[57] Granger C. B., McMurray J. J., Yusuf S. (2003). Effects of candesartan in patients with chronic heart failure and reduced left-ventricular systolic function intolerant to angiotensin-converting-enzyme inhibitors: the CHARM-Alternative trial. *Lancet*.

[58] Zou Y., Hu Y., Metzler B., Xu Q. (1998). Signal transduction in arteriosclerosis: mechanical stress-activated MAP kinases in vascular smooth muscle cells (review). *International Journal of Molecular Medicine*.

[59] Wang C., Qian X., Sun X., Chang Q. (2015). Angiotensin II increases matrix metalloproteinase 2 expression in human aortic smooth muscle cells via AT1R and ERK1/2. *Experimental Biology and Medicine*.

[60] Ji Y., Liu J., Wang Z., Liu N. (2009). Angiotensin II induces inflammatory response partly via toll-like receptor 4-dependent signaling pathway in vascular smooth muscle cells. *Cellular Physiology and Biochemistry*.

[61] Zhang Z., Yang Z., Wang S., Wang X., Mao J. (2024). Targeting MAPK-ERK/JNK pathway: a potential intervention mechanism of myocardial fibrosis in heart failure. *Biomedicine & Pharmacotherapy*.

[62] Dugourd C., Gervais M., Corvol P., Monnot C. (2003). Akt is a major downstream target of PI3-kinase involved in angiotensin II-induced proliferation. *Hypertension*.

[63] Pantan R., Tocharus J., Phatsara M., Suksamrarn A., Tocharus C. (2016). Synergistic effect of atorvastatin and cyanidin-3-glucoside Against angiotensin II-mediated vascular smooth muscle cell proliferation and migration through MAPK and PI3K/Akt pathways. *Archives of pharmacal research*.

[64] Zhao H., Li M., Wang L. (2012). Angiotensin II induces TSLP via an AT1 receptor/NF-KappaB pathway, promoting Th17 differentiation. *Cellular Physiology and Biochemistry*.

[65] Ruiz-Ortega M., Lorenzo O., Rupérez M., König S., Wittig B., Egido J. (2000). Angiotensin II activates nuclear transcription factor kappaB through AT (1) and AT(2) in vascular smooth muscle cells: molecular mechanisms. *Circulation Research*.

[66] He R., Zhang J., Luo D. (2019). Upregulation of transient receptor potential canonical type 3 channel via AT1R/TGF-*β*1/Smad 2/3 induces atrial fibrosis in aging and spontaneously hypertensive rats. *Oxidative Medicine and Cellular Longevity*.

[67] Yuan P., Liu J., Xiong S. (2023). Effects and mechanism of Compound Qidan Formula on rats with HFpEF induced by hypertension and diabetes mellitus based on Ang II/TGF-*β*1/Smads signaling pathway. *Journal of Ethnopharmacology*.

[68] Shimokawa H., Sunamura S., Satoh K. (2016). RhoA/Rho-kinase in the cardiovascular system. *Circulation Research*.

[69] Cao X., Luo T., Luo X., Tang Z. (2014). Resveratrol prevents AngII-induced hypertension via AMPK activation and RhoA/ROCK suppression in mice. *Hypertension Research*.

[70] Wynne B. M., Chiao C. W., Webb R. C. (2009). Vascular smooth muscle cell signaling mechanisms for contraction to angiotensin II and endothelin-1. *Journal of the American Society of Hypertension*.

[71] Jin L., Ying Z., Hilgers R. H. (2006). Increased RhoA/Rho-kinase signaling mediates spontaneous tone in aorta from angiotensin II-induced hypertensive rats. *The Journal of Pharmacology and Experimental Therapeutics*.

[72] Huang J., Qu Q., Dai Y., Ren D., Qian J., Ge J. (2023). Detrimental role of PDZ-RhoGEF in pathological cardiac hypertrophy. *Hypertension*.

