# Supplementary figures and images for: Imperatorin's Effect on Myocardial Infarction Based on Network Pharmacology and Molecular Docking
Source: Cardiovasc Ther. 2025 Jan 13;2025:7551459. doi: 10.1155/cdr/7551459 (PMC11745561; doi:10.1155/cdr/7551459)

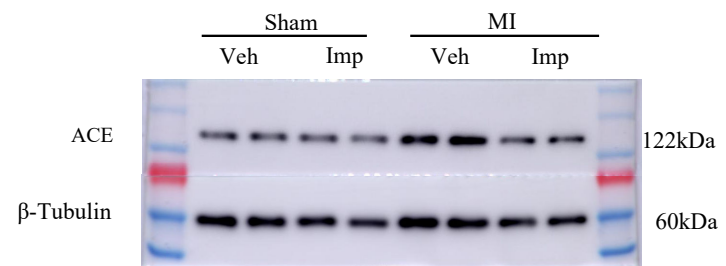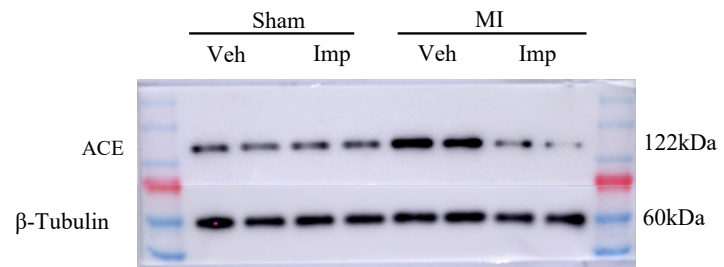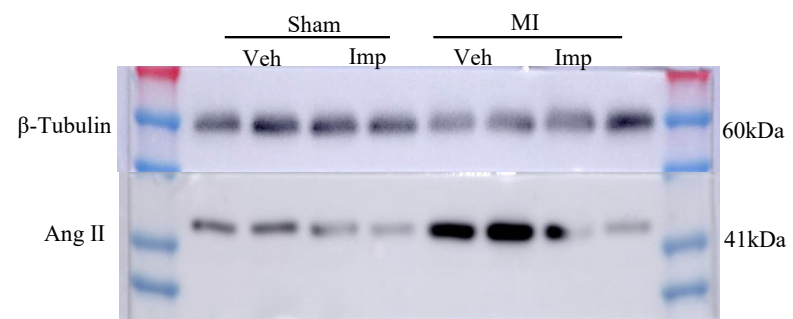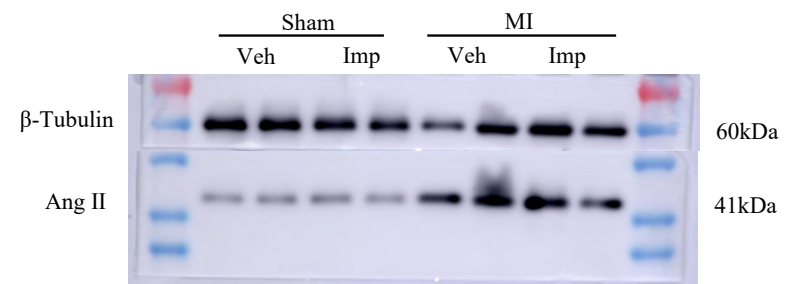

Supplement: Supporting Information — Additional supporting information can be found online in the Supporting Information section. The supporting information includes the original uncropped western blot image corresponding to Figure 9(b) in the main manuscript. This is provided as Figure S1 to ensure the transparency and reproducibility of the experimental data. [file 7551459.f1.pdf]
